# Comparison of treatment outcomes of direct oral anticoagulants and heparin for patients with Takotsubo cardiomyopathy: A nationwide cohort analysis

**DOI:** 10.1371/journal.pone.0336960

**Published:** 2025-11-13

**Authors:** Sadahiro Hijikata, Norihiko Inoue, Kiyohide Fushimi, Tetsuo Sasano

**Affiliations:** 1 Department of Cardiovascular Medicine, Graduate School of Medical and Dental Sciences, Institute of Science Tokyo, Tokyo, Japan; 2 Department of Health Policy and Informatics, Graduate School of Medical and Dental Sciences, Institute of Science Tokyo, Tokyo, Japan; 3 Department of Clinical Data Management and Research, Clinical Research Center, National Hospital Organization Headquarters, Tokyo, Japan; 4 Institute of Clinical Epidemiology (ICE), Showa University, Tokyo, Japan; Royal Holloway University of London, UNITED KINGDOM OF GREAT BRITAIN AND NORTHERN IRELAND

## Abstract

Takotsubo cardiomyopathy is characterized by temporary ballooning of the left ventricle and may lead to thrombosis, necessitating anticoagulation therapy in high-risk patients. However, no previous studies have compared anticoagulation therapies for this population. We aimed to compare the clinical outcomes of patients treated with direct oral anticoagulants (DOACs) and those treated with heparin. This retrospective study included patients with Takotsubo cardiomyopathy receiving DOACs or heparin within the first 2 days of admission between April 2012 and March 2021, identified from a nationwide in-hospital database in Japan. The primary outcome was in-hospital mortality. The secondary outcomes were ischemic and bleeding events, hospitalization costs, and length of hospital stay. After adjustment with propensity score-based inverse probability weighting, the risks of outcomes were estimated using Poisson regression models. Among 4,813 patients, 530 received DOACs and 4,283 received heparin. The DOAC group was older than the heparin group (mean [standard deviation] 78.1 [9.4] vs. 74.4 [11.2] years). After covariate adjustment, in-hospital mortality (4.0% vs. 3.8%; p = 0.87), ischemic events (1.1% vs. 2.8%; p = 0.067), and bleeding events (0.2% vs. 0.3%; p = 0.67) did not significantly differ between the DOAC and heparin groups. In contrast, the DOAC group had shorter hospital stays (median, 11 days vs. 13 days; p < 0.001) and lower total hospitalization costs ($5,181 USD vs. $6,084 USD; p = 0.003). These findings provide clinicians with valuable insights regarding the use of DOACs for patients with Takotsubo cardiomyopathy.

## Introduction

Takotsubo cardiomyopathy, named after the Japanese term for “octopus pot” because of its characteristic temporary ballooning of the left ventricle, was first described in 1990 [1]. Although initially considered a benign and reversible condition, it is currently recognized as a critical condition associated with life-threatening arrhythmias, systemic thromboembolism, heart failure, cardiac shock, and death [2–5]. It is estimated to occur in 1–4% of patients presenting with possible acute coronary syndrome, with a mortality rate of 3–6%, which is comparable to that of the syndrome [3–6]. During the first 30 days after admission, major adverse cardiac and cerebrovascular events occurred in 7.1% of cases, with stroke and transient ischemic attack being the most frequent [5]. Although the use of antithrombotic therapy has been discussed, no clinical research has specifically focused on Takotsubo cardiomyopathy [7–9]. According to an expert consensus of the European Society of Cardiology, anticoagulation with heparin is recommended for high-risk patients, including those with severely reduced ejection fraction and apical ballooning [9]. Direct oral anticoagulants (DOACs), including dabigatran, rivaroxaban, apixaban, and edoxaban, may serve as alternatives to heparin. Unlike heparin, DOACs do not require injections or routine monitoring [10], rendering them a more practical option. In addition, their oral administration, predictable anticoagulant effect without dose adjustment, and avoidance of prolonged intravenous access may simplify inpatient management and facilitate earlier discharge. However, evidence supporting the use of DOACs for Takotsubo cardiomyopathy is limited. Therefore, in this study, we compared the prognosis and clinical outcomes of patients treated with DOACs and those of patients treated with heparin using data of a Japanese inpatient cohort. Additionally, we assessed the efficacy and safety of each anticoagulation treatment strategy.

## Methods

This retrospective study used data extracted from a nationwide diagnostic and procedural combination (DPC) database, which is comparable to the diagnosis-related group payment system in the United States. It was initiated in 2002 as a nationwide reimbursement system for acute inpatient care in Japan and currently covers nearly all acute care hospitals in the country [2,11,12]. Diagnostic codes in the database are classified using the International Classification of Diseases, 10th revision (ICD-10), and grouped into the following six distinct categories: main disease, reason-for-admission diagnosis, most-consuming diagnosis, second-consuming diagnosis, comorbidities identified at admission, and complications arising after admission. Over 20 validation studies of algorithms using diagnoses and medication codes have been reported [13–15]. In particular, the validity of the algorithm for Takotsubo cardiomyopathy diagnosis using the database has been established with a positive predictive value of 1.00 [14]. Further details of the database used in this study are provided in the Supporting Information. This study adhered to the ethical guidelines outlined in the Declaration of Helsinki. The requirement for informed consent was waived because the database contains anonymous information. The data for this research were accessed on October 4, 2023. This study was approved by the Review Board of the Institute of Science Tokyo (M2000-788).

### Setting and participants

**Fig 1:**
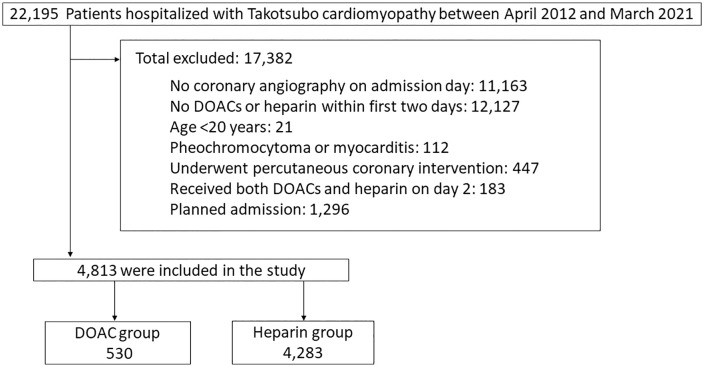
Study flow chart. DOAC, direct oral anticoagulant.

Patients diagnosed with Takotsubo cardiomyopathy between April 2012 and March 2021 were enrolled in this retrospective cohort study (Fig 1). Takotsubo cardiomyopathy cases were identified using ICD-10 code I51.8, extracted from the reason-for-admission diagnosis, main diagnosis, or most resource-consuming diagnosis category. Among them, patients who underwent coronary angiography on the day of admission and who were initiated on anticoagulation therapy with either DOACs or heparin within the first 2 days of admission and continued through day 2 were included. The exclusion criteria for this study were as follows: age younger than 20 years; a diagnosis of myocarditis (ICD-10 code I40) or pheochromocytoma (ICD-10 code D35.0) based on the Mayo Clinic diagnostic criteria [16]; planned admission; subsequent hospitalizations for Takotsubo cardiomyopathy after the index hospitalization; and undergoing percutaneous coronary intervention, as outlined in a previously validated study [14]. Additionally, patients receiving both DOACs and heparin on day 2 who could not be assigned to either treatment group were excluded. Subsequently, patients were classified into the DOAC and heparin groups based on the anticoagulant therapy received.

### Covariates

Demographic data of patients, including age, sex, body mass index, smoking status, and Barthel index, were collected at the time of admission to evaluate their activities of daily living scores [17]. Baseline comorbidities were selectively identified using the diagnostic columns labeled comorbidities identified at admission and reason-for-admission diagnosis. The Charlson comorbidity index was derived from the diagnostic codes [18]. Data on medication use were classified according to the World Health Organization Anatomical Therapeutic Chemical classification system, which was used to identify cases of hypertension (C08 and C09), diabetes mellitus (A10), and dyslipidemia (C10), as well as their corresponding diagnostic codes. Additionally, the use of beta-blockers (C07), antiplatelet drugs (B01AC), proton pump inhibitors (A02BC), and H2-receptor antagonists (A02BA) was documented. Data on the severity of the condition, including consciousness and use of inotropes (C01C), oxygen therapy, mechanical circulation support devices, and cardiopulmonary resuscitation, were also recorded. Furthermore, we collected information on whether patients were treated in intensive care units or admitted to hospitals certified by the Japanese Circulation Society as teaching hospitals. Data on baseline medication use and patient status were collected only within the first 2 days of hospitalization. Comprehensive details on the extraction of diagnostic and procedural codes are provided in the Supporting Information.

### Outcomes

The primary outcome was in-hospital mortality. The secondary outcomes were ischemic and bleeding events, blood transfusions, hospitalization length, and total hospitalization costs. Ischemic events were defined using a combination of specific diagnostic codes suggesting complications after admission, including cerebral infarction (ICD-10 code I63), transient ischemic attack (ICD-10 code G45), and arterial thrombosis (ICD-10 code I74), as well as relevant diagnostic imaging codes. Computed tomography and magnetic resonance imaging codes were used to confirm diagnoses of cerebral infarction or transient ischemic attack, whereas ultrasonography codes were included in the definition of arterial thrombosis [19,20]. Bleeding events included intracerebral hemorrhage and gastrointestinal bleeding. Intracerebral hemorrhage was defined by a combination of specific diagnostic codes (ICD-10 codes I60, I61, or I62), suggesting complications after admission, in conjunction with the corresponding computed tomography or magnetic resonance imaging codes. Gastrointestinal bleeding was defined using the following criteria [21]: the use of hemostatic techniques or devices; simultaneous endoscopy and hemostatic agent application; diagnosis and treatment of ruptured esophageal varices; and percutaneous arterial embolization accompanied by related diagnoses. These imaging and procedural codes were collected from day 2 onward when patients were grouped based on their antithrombotic treatment. Total hospitalization costs were estimated using the reference prices from the Japanese national fee schedule, along with item-by-item prices for surgical procedures, examinations, devices, rehabilitation, and medications [12]. Outcomes, except for total hospitalization length and cost, were assessed starting from this point.

### Statistical analysis

Continuous values are presented as means with standard deviations. Categorical values are presented as numbers and percentages, with differences assessed using the Chi-square tests or Fisher exact tests. A p-value <0.05 was considered statistically significant. Missing data for body mass index (8.7%), Barthel index (17.1%), and consciousness level scores (1.0%) were imputed using a random forest-based algorithm with the “missForest” package, incorporating all predictors and outcomes. This machine learning-based imputation method demonstrates superior validity compared to alternative approaches, such as k-nearest neighbor imputation or multivariate imputation with chained equations [22]. To address potential confounding factors, inverse probability of treatment weighting (IPTW) was conducted using propensity scores derived from logistic regression representing the probability of receiving each antithrombotic therapy based on all baseline covariates. The analysis included the average treatment effect on the treated framework, and the weight for patients who received DOACs was fixed as 1. The distribution of stabilized weights was examined. To reduce the influence of extreme values, weights were truncated at the 1st and 99th percentiles, as recommended by a prior methodological study [23]. The covariates were considered well-balanced when the standardized mean difference was < 0.10. Risk ratios (RRs) and their corresponding 95% confidence intervals (CIs) in the IPTW cohort were analyzed using Poisson regression. The difference in the distribution of the length of hospital stay and total hospitalization costs between the groups was analyzed using a gamma regression model with a log link to account for the skewed distribution of these variables [24]. Furthermore, 1:1 propensity score matching was applied for a sensitivity analysis to confirm the robustness of the findings, using a caliper of 0.2 standard deviations of the logit of the propensity score. To evaluate specific outcomes of patients with concomitant Takotsubo cardiomyopathy and atrial fibrillation, we performed a separate analysis of this cohort and re-estimated the propensity score model. All statistical analyses were conducted using R version 4.3.1 (R Foundation for Statistical Computing, Vienna, Austria).

## Results

### Patient characteristics

Data from 22,195 hospitalized patients were assessed using the DPC database. After exclusion criteria were applied, 4,813 patients were eligible for the analysis, including 530 in the DOAC group and 4,283 in the heparin group (Fig 1). The baseline characteristics are presented in Table 1. Patients in the DOAC group were older, with a mean (standard deviation) age of 78.1 (9.4) years, than those in the heparin group (74.4 [11.2] years). The prevalence of atrial fibrillation differed between the groups, at 53.2% in the DOAC group and 7.0% in the heparin group. Patients in the DOAC group required fewer medical interventions, such as inotropes, oxygen therapy, mechanical ventilation, and intensive care unit admission, than those in the heparin group. After IPTW, the standardized mean difference of baseline characteristics was < 0.1, indicating an adequate balance between groups.

**Table 1:** Patient characteristics before and after IPTW.

	Before IPTW			After IPTW		
	DOAC	Heparin	SMD	DOAC	Heparin	SMD
n	530	4283				
Age, years, mean (SD)	78.1 (9.4)	74.4 (11.2)	0.353	78.1 (9.4)	77.8 (9.6)	0.029
Female sex	421 (79.4)	3574 (83.4)	0.103	79.4	80.4	0.024
BMI, kg/m^2^, mean (SD)	21.3 (3.8)	20.9 (3.7)	0.082	21.3 (3.8)	21.2 (3.7)	0.007
Smoker	86 (16.2)	781 (18.2)	0.053	16.2	16.3	0.001
Barthel Index			0.167			0.037
Totally dependent [0–20]	187 (41.3)	1749 (49.4)		38.3	39.9	
Partially dependent [25–95]	104 (23.0)	666 (18.8)		25.1	23.8	
Independent [100]	162 (35.8)	1122 (31.7)		36.6	36.4	
Charlson score			0.097			0.029
0	122 (23.0)	1018 (23.8)		23.0	22.2	
1	222 (41.9)	1638 (38.2)		41.9	42.2	
2	132 (24.9)	1080 (25.2)		24.9	24.7	
3 or more	54 (10.2)	547 (12.8)		10.2	10.9	
Hypertension	364 (68.7)	2478 (57.9)	0.226	68.7	66.2	0.054
Diabetes mellitus	109 (20.6)	830 (19.4)	0.03	20.6	20.1	0.012
Dyslipidemia	202 (38.1)	1507 (35.2)	0.061	38.1	36.1	0.041
Atrial fibrillation	282 (53.2)	301 (7.0)	1.165	53.2	48.6	0.092
Pulmonary embolism	4 (0.8)	15 (0.4)	0.055	0.8	0.9	0.016
Deep vein thrombosis	14 (2.6)	36 (0.8)	0.138	2.6	3.0	0.021
Cerebrovascular disease	10 (1.9)	73 (1.7)	0.014	1.9	1.7	0.011
Severe kidney disease	0 (0.0)	52 (1.2)	0.157	0.0	0.0	0
Malignancy	28 (5.3)	232 (5.4)	0.006	5.3	5.3	0.001
Metastatic malignancy	3 (0.6)	31 (0.7)	0.02	0.6	0.8	0.025
COPD	8 (1.5)	123 (2.9)	0.093	1.5	1.3	0.017
Sepsis	1 (0.2)	32 (0.7)	0.082	0.2	0.2	0.011
Inflammation disease	127 (24.0)	1206 (28.2)	0.096	24.0	25.1	0.026
Trigger			0.171			0.034
Physical	174 (32.8)	1758 (41.0)		32.8	34.4	
Emotional or unknown	3 (0.6)	66 (1.5)		67.2	65.6	
Heart failure	243 (45.8)	1753 (40.9)	0.099	45.8	46.6	0.015
Japan Coma Scale			0.103			0.032
Alert [0]	442 (84.7)	3480 (82.0)		84.7	83.5	
Dizziness [1 –3 ]	61 (11.7)	553 (13.0)		11.7	12.5	
Somnolence [10–30]	12 (2.3)	98 (2.3)		2.3	2.5	
Coma [100–300]	7 (1.3)	111 (2.6)		1.3	1.5	
Cardiopulmonary resuscitation	5 (0.9)	58 (1.4)	0.039	0.9	0.7	0.023
IABP	1 (0.2)	127 (3.0)	0.224	0.2	0.2	0.004
ECMO or microaxial flow pump	0 (0.0)	21 (0.5)	0.205	0.0	0.0	0
Mechanical ventilation	23 (4.3)	503 (11.7)	0.275	4.3	5.8	0.068
Oxygen therapy	270 (50.9)	2453 (57.3)	0.127	50.9	53.0	0.041
Intensive care unit	78 (14.7)	860 (20.1)	0.142	14.7	14.6	0.003
JCS certified hospital	215 (40.6)	1887 (44.1)	0.071	40.6	38.6	0.04
Inotrope	87 (16.4)	1074 (25.1)	0.215	16.4	18.1	0.044
Beta-blocker	209 (39.4)	941 (22.0)	0.386	39.4	35.3	0.085
Antiplatelet drug	245 (46.2)	2098 (49.0)	0.055	46.2	44.9	0.027
Warfarin	1 (0.2)	309 (7.2)	0.379	0.2	0.2	0.006
Proton pump inhibitor	285 (53.8)	1923 (44.9)	0.178	53.8	49.9	0.077
Histamin-2 receptor antagonist	26 (4.9)	359 (8.4)	0.14	4.9	5.6	0.03
Dabigatran	25 (4.7)			4.7		
Rivaroxaban	108 (20.4)			20.4		
Apixaban	190 (35.8)			35.8		
Edoxaban	209 (39.4)			39.4		
Unfractionated heparin		4283 (100.0)			100.0	
Low-molecular-weight heparin		2 (0.0)			0.0	

Categorical variables are presented as n (%) before IPTW and weighted percentages after IPTW. Age and BMI are presented as means (standard deviation). BMI, body mass index; COPD, chronic obstructive pulmonary disease; DOAC, direct oral anticoagulant; ECMO, extracorporeal membrane oxygenation; IABP, intra-aortic balloon pump; IPTW, inverse probability of treatment weighting; JCS, Japan Circulation Society; SD, standard deviation; SMD, standardized mean difference.

### Outcomes

The clinical outcomes of patients are presented in Table 2. After IPTW, the mortality rates were 4.0% in the DOAC group and 3.8% in the heparin group (RR 1.05 [95% CI 0.59–1.88], p = 0.87). The rates of ischemic events were 1.1% in the DOAC group and 2.8% in the heparin group (RR, 0.41 [95% CI 0.15–1.07], p = 0.067). The rate of bleeding events was 0.2% and 0.3% in the DOAC and heparin groups, respectively. The blood transfusion rate was lower in the DOAC group at 1.9%, compared with the heparin group at 5.4% (RR 0.35 [95% CI 0.17–0.69], p = 0.003). The length of hospitalization and associated costs are presented in Table 3. After IPTW was applied, the length of hospital stay was significantly shorter in the DOAC group (median, 11 days; interquartile range. [IQR], 8–17 days) than in the heparin group (median, 13 days; IQR, 9–19 days) (p < 0.001). The total hospitalization cost was significantly lower in the DOAC group (median, $5,181 USD; IQR, $4,000–$7,640 USD) than in the heparin group ($6,084 USD; IQR, $4,536–$8,299 USD) (p = 0.003).

**Table 2:** Clinical outcomes of the DOAC and heparin groups.

	Before IPTW		After IPTW			
	DOAC	Heparin	DOAC	Heparin	RR	
n	530	4283			[95% CI]	p
Primary outcome						
In-hospital mortality	21 (4.0)	174 (4.1)	4.0	3.8	1.05 [0.59-1.88]	0.87
						
Secondary outcomes						
Ischemic events	6 (1.1)	59 (1.4)	1.1	2.8	0.41 [0.15-1.07]	0.067
Cerebral infarction	6 (1.1)	45 (1.1)	1.1	2.6	0.43 [0.16-1.16]	0.096
Transient ischemic attack	0 (0.0)	2 (0.0)	0.0	0.0	NA	NA
Arterial thrombosis	0 (0.0)	12 (0.3)	0.0	0.2	NA	NA
Bleeding events	1 (0.2)	24 (0.6)	0.2	0.3	0.59 [0.07-4.76]	0.62
Intracranial hemorrhage	0 (0.0)	11 (0.3)	0.0	0.1	NA	NA
Gastrointestinal bleeding	1 (0.2)	13 (0.3)	0.2	0.2	0.85 [0.09-7.72]	0.89
Blood transfusion	10 (1.9)	239 (5.6)	1.9	5.4	0.35 [0.17-0.69]	0.003

All outcomes are presented as n (%) before IPTW and weighted percentages after IPTW. The RRs and CIs were not calculated if one group had no events for a specific outcome. CI, confidence interval; DOAC, direct oral anticoagulant; IPTW, inverse probability of treatment weighting; NA, not applicable; RR, risk ratio.

**Table 3:** Hospitalization lengths and costs for the DOAC and heparin groups.

	Before IPTW		After IPTW		
	DOAC	Heparin	DOAC	Heparin	p
n	530	4283			
Length of hospital stay (days)	11 (8–17)	13 (9–19)	11 (8–17)	13 (9–19)	<0.001
Total hospitalization cost (USD)	5190 (4000–7623)	6130 (4569–-9013)	5181 (4000–7640)	6084 (4536–8299)	0.003

Data are presented as median (interquartile range, Q1–Q3). DOAC, direct oral anticoagulant; IPTW, inverse probability of treatment weighting.

### Additional analyses

To confirm the robustness of our results, a sensitivity analysis was performed using 1:1 propensity score matching (S1, S2, and S3 Tables). Baseline covariates exhibited a well-balanced distribution between the DOAC and heparin groups. The sensitivity analysis showed similar results to those of the IPTW cohort. No significant differences were observed in the rates of mortality, ischemic events, and bleeding events between groups. The DOAC group had a significantly lower blood transfusion rate, shorter hospitalization period, and lower total hospitalization costs than the heparin group. Additionally, the findings of a separate analysis restricted to the cohort of patients with atrial fibrillation were consistent with those of the primary analysis (S4 and S5 Tables).

## Discussion

We compared the clinical outcomes of anticoagulation therapies for high-risk patients with Takotsubo cardiomyopathy. To our knowledge, this is the first study to compare the efficacy and safety of DOACs and heparin in this population. In this study, the DOAC and heparin groups had comparable in-hospital mortality. The risk ratio for ischemic events was 0.41, suggesting a trend towards a lower risk in the DOAC group; however, this difference was not statistically significant. Although both groups had low bleeding event rates, the blood transfusion rate in the DOAC group was significantly lower than that in the heparin group. The propensity score matching analysis and separate analysis of the cohort of patients with atrial fibrillation revealed results consistent with those of the primary analysis. Our findings suggest that DOACs may serve as alternatives to heparin for patients with Takotsubo cardiomyopathy. Compared with heparin, DOACs offer the advantages of not requiring dose adjustment based on activated partial thromboplastin time or prolonged intravenous administration throughout hospitalization [10]. These factors may enable earlier discharge of patients on DOACs, thereby potentially reducing hospitalization costs.

In conditions comparable to severely reduced left ventricle wall motion, such as myocardial infarction and dilated cardiomyopathy, studies on the prevention of left ventricle thrombosis have compared the use of DOACs with warfarin, a traditional oral anticoagulant [25–27]. In a meta-analysis involving 824 patients treated with DOACs and 2,233 patients treated with warfarin, Levine et al. reported no significant differences in mortality, ischemic events, or bleeding complications between the two groups [25], indicating that DOACs may be a comparable option to warfarin.

Antithrombotic therapy with heparin is recommended by the European Society of Cardiology expert consensus for patients with Takotsubo cardiomyopathy classified as high-risk for left ventricle thrombosis, including those with low ejection fraction or apical ballooning type [9]. Recent data from the Swedish national population registry indicated that the use of heparin in patients with Takotsubo cardiomyopathy was associated with reduced 30-day mortality, whereas the use of oral anticoagulants was not significantly correlated with reduced mortality [28]. In contrast, our study adjusted for the timing of anticoagulant initiation and covariates, an approach not applied in the Swedish registry study.

Inflammation plays an important role in the pathogenesis of Takotsubo cardiomyopathy [29]. Ding et al. reported that patients with Takotsubo cardiomyopathy who experienced thrombotic events exhibited significantly elevated inflammatory markers, including C-reactive protein and white blood cell counts [30]. Jafri et al. revealed that heparin has a potential role in reducing pro-inflammatory cytokines, such as interleukin-6 and interleukin-8 [31], whose levels are significantly elevated in Takotsubo cardiomyopathy [32]. These findings suggest an alternative role of heparin as an anticoagulant. Therefore, the comparison between DOACs and heparin remains a critical area of research owing to their different mechanisms and potential benefits.

This study has important strengths in comparing the efficacy and safety of DOACs and heparin in patients with Takotsubo cardiomyopathy. First, we enrolled a cohort of 4,813 patients from nearly all acute care hospitals in Japan, based on the high validity of Takotsubo cardiomyopathy diagnosis in the database. Second, our study design adjusted for the timing of anticoagulant initiation and other covariates, which minimized selection bias. Finally, we applied IPTW to balance baseline characteristics between the DOAC and heparin groups.

However, this study has several limitations. First, the DPC database used in this study did not include detailed laboratory findings or echocardiographic data, such as Takotsubo cardiomyopathy types and left ventricular ejection fraction, or the presence of a thrombus at diagnosis. Furthermore, other potentially influential acute physiological parameters, including precise blood pressure values, detailed arrhythmia characteristics, more granular assessments of overall disease severity, and temporal changes in treatment patterns, were not collected in the database. Therefore, the potential for residual confounding attributable to these specified factors and other unrecognized variables was an important limitation of this study. Second, the accuracy of comorbidity diagnoses and nonfatal outcomes in the database may have been affected by coding errors or misclassifications. Third, the number of patients in the DOAC group differed from that in the heparin group. Although no significant differences in ischemic events were observed between groups, the possibility of insufficient statistical power cannot be ruled out. Fourth, nonfatal outcomes may have been affected by competing risks. Fifth, the database did not include information on pre-admission medications; thus, prior anticoagulant use could not be assessed. Sixth, indication bias was a significant concern, particularly because of the high prevalence of atrial fibrillation in the DOAC group. Because the DPC database does not capture the specific dosing or indication for each prescription, residual indication bias remains a significant concern despite adjustments for baseline diagnoses and other measured comorbidities. A direct comparison of anticoagulation durations in the treatment groups was not appropriate because differences in these durations likely reflected underlying variations in treatment indications and baseline characteristics. Additionally, unfractionated heparin was predominantly used due to limited indications and reimbursement constraints for low-molecular-weight heparin in Japan. As unfractionated heparin is associated with a higher bleeding risk, this may limit the generalizability of our findings. Finally, the DPC data contain only in-hospital outcomes. This limitation is particularly relevant to the shorter hospital stays in the DOAC group, as post-discharge events such as mortality or hospital readmissions could not be assessed. Therefore, further research including pre-hospital information, echographic data, morphological variants of Takotsubo cardiomyopathy, and post-discharge outcomes is needed to validate the findings and provide a more comprehensive understanding of the effect of anticoagulants on patient prognoses.

## Conclusion

In this study, the efficacy and safety outcomes of DOACs were not significantly different from those of heparin among patients with Takotsubo cardiomyopathy who required anticoagulation. However, the DOAC group required fewer healthcare resources than the heparin group. Our findings suggest that DOACs may serve as a viable alternative to heparin for patients with Takotsubo cardiomyopathy; however, further research is required to validate these results and explore long-term outcomes.

## Supporting information

S1 File Details of the DPC database. (DOCX)

S2 File Definition of inclusion and exclusion criteria. (DOCX)

S3 File Definition of baseline characteristics of comorbidities at admission. (DOCX)

S4 File Definitions of outcomes. (DOCX)

S1 Table Patient characteristics after propensity score matching. Data are presented as n (%). Age and BMI are presented as mean (standard deviation). Continuous variables were compared using the t-test and categorical variables were compared using the chi-squared test. BMI, body mass index; COPD, chronic obstructive pulmonary disease; IABP, intra-aortic balloon pump; ECMO, extracorporeal membrane oxygenation; JCS, Japan Circulation of Society.
(DOCX)

S2 Table Clinical outcomes in the matched cohort. DOAC, direct oral anticoagulant; NA; not available; RR, incidence rate ratio.
(DOCX)

S3 Table Length of hospital stay and hospitalization costs in the matched cohort. DOAC, direct oral anticoagulant.
(DOCX)

S4 Table Patient characteristics in the atrial fibrillation cohort before and after IPTW. Categorical variables are presented as n (%) before IPTW and as weighted percentages after IPTW. Age and BMI are presented as means (standard deviation). BMI, body mass index; COPD, chronic obstructive pulmonary disease; DOAC, direct oral anticoagulant; ECMO, extracorporeal membrane oxygenation; IABP, intra-aortic balloon pump; IPTW, inverse probability of treatment weighting; JCS, Japan Circulation Society; SMD, standardized mean difference.
(DOCX)

S5 Table Clinical outcomes in the atrial fibrillation cohort. All outcomes are presented as n (%) before IPTW and as weighted percentages after IPTW. The RRs and CIs were not calculated if one group had no events for a specific outcome. CI, confidence interval; DOAC, direct oral anticoagulant; IPTW, inverse probability of treatment weighting; NA, not applicable; RR, risk ratio.
(DOCX)

## References

[1] Sato H, Tateishi H, Uchida T, Dote K, Ishihara M. Tako-tsubo like cardiomyopathy due to multivessel spasm. Clinical aspect of myocardial injury: From ischemia to heart failure. Tokyo: Kagakuhyoronsha Co. 1990:56–64.

[2] Isogai T, Matsui H, Tanaka H, Makito K, Fushimi K, Yasunaga H. Incidence, management, and prognostic impact of arrhythmias in patients with Takotsubo syndrome: a nationwide retrospective cohort study. Eur Heart J Acute Cardiovasc Care. 2023;12(12):834–46. doi: 10.1093/ehjacc/zuad110 37708494 PMC10734680

[3] Stiermaier T, Eitel C, Denef S, Desch S, Schuler G, Thiele H, et al. Prevalence and Clinical Significance of Life-Threatening Arrhythmias in Takotsubo Cardiomyopathy. J Am Coll Cardiol. 2015;65(19):2148–50. doi: 10.1016/j.jacc.2015.02.062 25975480

[4] Vallabhajosyula S, Barsness GW, Herrmann J, Anavekar NS, Gulati R, Prasad A. Comparison of Complications and In-Hospital Mortality in Takotsubo (Apical Ballooning/Stress) Cardiomyopathy Versus Acute Myocardial Infarction. Am J Cardiol. 2020;132:29–35. doi: 10.1016/j.amjcard.2020.07.015 32762963

[5] Templin C, Ghadri JR, Diekmann J, Napp LC, Bataiosu DR, Jaguszewski M, et al. Clinical Features and Outcomes of Takotsubo (Stress) Cardiomyopathy. N Engl J Med. 2015;373(10):929–38. doi: 10.1056/NEJMoa1406761 26332547

[6] Arao K, Yoshikawa T, Isogai T, Imori Y, Mochizuki H, Sakata K, et al. A study of takotsubo syndrome over 9 years at the Tokyo Cardiovascular Care Unit Network Registry. J Cardiol. 2023;82(2):93–9. doi: 10.1016/j.jjcc.2022.12.011 36640906

[7] Medina deChazal H, Del Buono MG, Keyser-Marcus L, Ma L, Moeller FG, Berrocal D, et al. Stress Cardiomyopathy Diagnosis and Treatment: JACC State-of-the-Art Review. J Am Coll Cardiol. 2018;72(16):1955–71. doi: 10.1016/j.jacc.2018.07.072 30309474 PMC7058348

[8] Santoro F, Stiermaier T, Tarantino N, De Gennaro L, Moeller C, Guastafierro F, et al. Left Ventricular Thrombi in Takotsubo Syndrome: Incidence, Predictors, and Management: Results From the GEIST (German Italian Stress Cardiomyopathy) Registry. J Am Heart Assoc. 2017;6(12):e006990. doi: 10.1161/JAHA.117.006990 29203578 PMC5779019

[9] Ghadri J-R, Wittstein IS, Prasad A, Sharkey S, Dote K, Akashi YJ, et al. International Expert Consensus Document on Takotsubo Syndrome (Part II): Diagnostic Workup, Outcome, and Management. Eur Heart J. 2018;39(22):2047–62. doi: 10.1093/eurheartj/ehy077 29850820 PMC5991205

[10] Hirsh J, Bauer KA, Donati MB, Gould M, Samama MM, Weitz JI. Parenteral anticoagulants: American College of Chest Physicians Evidence-Based Clinical Practice Guidelines (8th Edition). Chest. 2008;133(6 Suppl):141S–159S. doi: 10.1378/chest.08-0689 18574264

[11] Hayashida K, Murakami G, Matsuda S, Fushimi K. History and Profile of Diagnosis Procedure Combination (DPC): Development of a Real Data Collection System for Acute Inpatient Care in Japan. J Epidemiol. 2021;31(1):1–11. doi: 10.2188/jea.JE20200288 33012777 PMC7738645

[12] Yasunaga H. Real world data in Japan: chapter II the diagnosis procedure combination Database. Ann Clin Epidemiol. 2019;(1):76–9.

[13] Yamana H, Konishi T, Yasunaga H. Validation studies of Japanese administrative health care data: A scoping review. Pharmacoepidemiol Drug Saf. 2023;32(7):705–17. doi: 10.1002/pds.5636 37146098

[14] Kanaoka K, Okayama S, Terasaki S, Nakano T, Ishii M, Nakai M, et al. Role of climatic factors in the incidence of Takotsubo syndrome: A nationwide study from 2012 to 2016. ESC Heart Fail. 2020;7(5):2629–36. doi: 10.1002/ehf2.12843 32715646 PMC7524088

[15] Hara K, Tomio J, Svensson T, Ohkuma R, Svensson AK, Yamazaki T. Association measures of claims-based algorithms for common chronic conditions were assessed using regularly collected data in Japan. J Clin Epidemiol. 2018;99:84–95. doi: 10.1016/j.jclinepi.2018.03.004 29548842

[16] Prasad A, Lerman A, Rihal CS. Apical ballooning syndrome (Tako-Tsubo or stress cardiomyopathy): a mimic of acute myocardial infarction. Am Heart J. 2008;155(3):408–17. doi: 10.1016/j.ahj.2007.11.008 18294473

[17] Mahoney FI, Barthel DW. Functional Evaluation: The Barthel Index. Md State Med J. 1965;14:61–5. 14258950

[18] Quan H, Li B, Couris CM, Fushimi K, Graham P, Hider P, et al. Updating and validating the Charlson comorbidity index and score for risk adjustment in hospital discharge abstracts using data from 6 countries. Am J Epidemiol. 2011;173(6):676–82. doi: 10.1093/aje/kwq433 21330339

[19] Tanigawa M, Kohama M, Nonaka T, Saito A, Tamiya A, Nomura H, et al. Validity of identification algorithms combining diagnostic codes with other measures for acute ischemic stroke in MID-NET®. Pharmacoepidemiol Drug Saf. 2022;31(5):524–33. doi: 10.1002/pds.5423 35224801

[20] Koretsune Y, Yamashita T, Yasaka M, Oda E, Matsubayashi D, Ota K, et al. Usefulness of a healthcare database for epidemiological research in atrial fibrillation. J Cardiol. 2017;70(2):169–79. doi: 10.1016/j.jjcc.2016.10.015 28027833

[21] Fukasawa T, Seki T, Nakashima M, Kawakami K. Comparative effectiveness and safety of edoxaban, rivaroxaban, and apixaban in patients with venous thromboembolism: A cohort study. J Thromb Haemost. 2022;20(9):2083–97. doi: 10.1111/jth.15799 35748327

[22] Stekhoven DJ, Bühlmann P. MissForest--non-parametric missing value imputation for mixed-type data. Bioinformatics. 2012;28(1):112–8. doi: 10.1093/bioinformatics/btr597 22039212

[23] Austin PC, Stuart EA. Moving towards best practice when using inverse probability of treatment weighting (IPTW) using the propensity score to estimate causal treatment effects in observational studies. Stat Med. 2015;34(28):3661–79. doi: 10.1002/sim.6607 26238958 PMC4626409

[24] Dodd S, Bassi A, Bodger K, Williamson P. A comparison of multivariable regression models to analyse cost data. J Eval Clin Pract. 2006;12(1):76–86. doi: 10.1111/j.1365-2753.2006.00610.x 16422782

[25] Levine GN, McEvoy JW, Fang JC, Ibeh C, McCarthy CP, Misra A, et al. Management of Patients at Risk for and With Left Ventricular Thrombus: A Scientific Statement From the American Heart Association. Circulation. 2022;146(15):e205–23. doi: 10.1161/CIR.0000000000001092 36106537

[26] Alcalai R, Butnaru A, Moravsky G, Yagel O, Rashad R, Ibrahimli M, et al. Apixaban vs. warfarin in patients with left ventricular thrombus: a prospective multicentre randomized clinical trial‡. Eur Heart J Cardiovasc Pharmacother. 2022;8(7):660–7. doi: 10.1093/ehjcvp/pvab057 34279598

[27] Huang L, Tan Y, Pan Y. Systematic review of efficacy of direct oral anticoagulants and vitamin K antagonists in left ventricular thrombus. ESC Heart Fail. 2022;9(5):3519–32. doi: 10.1002/ehf2.14084 35894752 PMC9715875

[28] Petursson P, Oštarijaš E, Redfors B, Råmunddal T, Angerås O, Völz S, et al. Effects of pharmacological interventions on mortality in patients with Takotsubo syndrome: a report from the SWEDEHEART registry. ESC Heart Fail. 2024;11(3):1720–9. doi: 10.1002/ehf2.14713 38454651 PMC11098647

[29] Singh T, Khan H, Gamble DT, Scally C, Newby DE, Dawson D. Takotsubo Syndrome: Pathophysiology, Emerging Concepts, and Clinical Implications. Circulation. 2022;145(13):1002–19. doi: 10.1161/CIRCULATIONAHA.121.055854 35344411 PMC7612566

[30] Ding KJ, Cammann VL, Szawan KA, Stähli BE, Wischnewsky M, Di Vece D, et al. Intraventricular Thrombus Formation and Embolism in Takotsubo Syndrome: Insights From the International Takotsubo Registry. Arterioscler Thromb Vasc Biol. 2020;40(1):279–87. doi: 10.1161/ATVBAHA.119.313491 31766870

[31] Jafri M, Li L, Liang B, Luo M. The Effect of Heparin and Other Exogenous Glycosaminoglycans (GAGs) in Reducing IL-1β-Induced Pro-Inflammatory Cytokine IL-8 and IL-6 mRNA Expression and the Potential Role for Reducing Inflammation. Pharmaceuticals (Basel). 2024;17(3):371. doi: 10.3390/ph17030371 38543157 PMC10976005

[32] Scally C, Abbas H, Ahearn T, Srinivasan J, Mezincescu A, Rudd A, et al. Myocardial and Systemic Inflammation in Acute Stress-Induced (Takotsubo) Cardiomyopathy. Circulation. 2019;139(13):1581–92. doi: 10.1161/CIRCULATIONAHA.118.037975 30586731 PMC6438459

