# Supplementary material for: Comparison of treatment outcomes of direct oral anticoagulants and heparin for patients with Takotsubo cardiomyopathy: A nationwide cohort analysis
Source: PLoS One. 2025 Nov 13;20(11):e0336960. doi: 10.1371/journal.pone.0336960 (PMC12614514; doi:10.1371/journal.pone.0336960)
Supplement: S1 File — (DOCX) [file pone.0336960.s001.docx]

**S1. Details of the DPC database**

The Diagnosis Procedure Combination (DPC) database is a comprehensive inpatient database that includes patient demographics, diagnostic codes, and procedural information of patients treated at almost all acute care hospitals in Japan. [1, 2]

|  |  |
| --- | --- |
| Diagnoses | The diagnostic codes in DPC database are categorized into six distinct parts: “main disease”, “reason-for-admission diagnosis”, “most-consuming diagnosis”, “second-consuming diagnosis”, “comorbidities already identified on admission, and “complications after admission”. These categories are described by the International Classification of Diseases, 10th revision (ICD-10) codes and Japanese standard disease code. |
| Hospital data | unique identifiers of the hospitals |
| Patient demographics | type of admission (urgent or elective) |
|  | age, sex, body weight, height, smoking index, pregnancy, discharge status, dates of admission and discharge, Barthel index |
| Clinical data | Japan coma scale |
| Administrative claims data | Pharmaceuticals and device |
|  | dates of procedures, drugs and devices |
|  | estimated costs |

Reference:

1. Hayashida K, Murakami G, Matsuda S, Fushimi K. History and Profile of Diagnosis Procedure Combination (DPC): Development of a Real Data Collection System for Acute Inpatient Care in Japan. J Epidemiol. 2021;31(1):1-11. Epub 20201121. doi: 10.2188/jea.JE20200288. PubMed PMID: 33012777; PubMed Central PMCID: PMCPMC7738645.

2. Yasunaga H. Real world data in Japan: chapter II the diagnosis procedure combination

database. Ann Clin Epidemiol. 2019;(1):76–9.
