# Supplementary material for: Comparison of treatment outcomes of direct oral anticoagulants and heparin for patients with Takotsubo cardiomyopathy: A nationwide cohort analysis
Source: PLoS One. 2025 Nov 13;20(11):e0336960. doi: 10.1371/journal.pone.0336960 (PMC12614514; doi:10.1371/journal.pone.0336960)
Supplement: S2 File — (DOCX) [file pone.0336960.s002.docx]

**S2. Definition of inclusion and exclusion criteria**

|  | ICD-10 code and Procedure code |
| --- | --- |
| **Inclusion** | |
| Patients with Takotsubo cardiomyopathy (I51.8) recorded as: “reason-for-admission diagnosis”, “main disease”, or “most-consuming diagnosis” in the national inpatient database of Japan from April 2012 to March 2022 were included. | |
| **Exclusion** | |
| Patients who met following condition or diagnosis codes identified as “comorbidities already identified on admission” or “admission-precipitating diagnosis” were excluded from this study. | |
| Age | Patients aged under 20 years at the time of admission were excluded. |
| Pheochromocytoma, myocarditis | Patients with pheochromocytoma or myocarditis were excluded, based on the Mayo Clinic diagnostic criteria.  *pheochromocytoma: ICD-10 code D35.0*  *myocarditis: ICD-10 code I40* |
| Coronary angiography | Patients who did not undergo coronary angiography (CAG) on the first day of admission were excluded.  *coronary angiography code: 160064510, 170020710* |
| Percutaneous coronary intervention | Patients who underwent percutaneous coronary intervention (PCI) were excluded.  *160107550, 150153910, 150260350, 150263310, 150284310, 150318310, 150359310, 150374910, 150375010, 150375110, 150375210, 150375310, 150375410, 150443750, 160107550, 726800000, 710010018, 710010019, 710010026, 710011014, 710011016* |
| Planned admission | Patients admitted electively were excluded. |
| DOAC and heparin | Patients who did not meet the anticoagulant criteria (initiating on anticoagulation therapy with either DOACs or heparin (≥5000 international units) within the first 2 days of hospitalization and continuing through day 2)  *ATC codes of DOACs: B01AE07 (dabigatran), B01AF01 (rivaroxaban), B01AF02 (apixaban), B01AF03 (edoxaban)*  *ATC codes of heparin: B01AB01(unfractionated heparin), B01AB04-12 (low-molecular weight heparin)* |

DOAC, direct oral anticoagulant; ICD-10, the International Classification of Diseases, 10th revision.
