# Supplementary material for: Comparison of treatment outcomes of direct oral anticoagulants and heparin for patients with Takotsubo cardiomyopathy: A nationwide cohort analysis
Source: PLoS One. 2025 Nov 13;20(11):e0336960. doi: 10.1371/journal.pone.0336960 (PMC12614514; doi:10.1371/journal.pone.0336960)
Supplement: S3 File — (DOCX) [file pone.0336960.s003.docx]

**S3. Definition of baseline characteristics of comorbidities at admission**

|  |  |
| --- | --- |
| Barthel Index[1] | The Barthel Index is a scale used to measure the performance in activities of daily living. It evaluates activities such as feeding, bathing, grooming, dressing, bowel control, bladder control, toilet use, transferring, mobility, and stair climbing. Scores were calculated upon admission and divided into three groups in this study: totally dependent (0–20), partially dependent (25–95), and independent (100). |
| Charlson score[2] | The Charlson Comorbidity Index categorizes 19 medical conditions to assess mortality risk. Conditions, including myocardial infarction, congestive heart failure, peripheral vascular disease, cerebrovascular disease, dementia, chronic pulmonary disease, rheumatologic disease, peptic ulcer disease, mild liver disease, and controlled diabetes mellitus, are assigned 1 point each. Hemiplegia, paraplegia, renal disease, localized malignancy, leukemia, lymphoma, and uncontrolled diabetes mellitus carry 2 points each. Moderate/severe liver disease is valued at 3 points. Metastatic tumor and AIDS are scoring 6 points. For analysis, the total scores calculated from the Charlson Comorbidity Index are categorized into four groups: 0 points, 1 point, 2 points, and 3 or more points. |
| Hypertension | *ICD-10 code: I10-I15*  **and/or**  Prescription for hypertension medications (calcium channel blockers – *ATC code C08*, agents acting on the renin-angiotensin system – *ATC code C09*) within the first two days of admission. |
| Diabetes mellitus | *ICD-10 code: E10-E14*  **and/or**  drugs for diabetes mellitus (*ATC code A10*) within the first two days of admission |
| Dyslipidemia | *ICD-10 code: E78*  **and/or**  drugs for lipid modifying agent (*ATC code C10*) within the first two days of admission |
| Atrial fibrillation | *ICD-10 code: I48* |
| Pulmonary embolism | *ICD-10 code: I26* |
| Venous thrombosis | *ICD-10 code: I80, O22, O87, O88* |
| Cerebrovascular disease | *ICD-10 code: I63* |
| Severe kidney disease | *ICD-10 code: N18.5, Z99.2*  **and/or** procedural code of dialysis  *140007710, 140007810, 140007910, 140008170, 140008410, 140008510, 140008670, 140008770, 140008810, 140036710, 140036810, 140051010, 140051110, 140052810, 140057810, 140057910, 140058010, 140058110, 140058210, 140058310, 140058410, 140058510, 140058610, 140058770, 140058870, 140058970, 140059070, 140059170, 140059270, 140059310, 140059410, 140059510, 140060210, 140060310, 140060410, 140060510, 140060610, 140060710, 140060810, 140060910, 140061010, 140037910, 140038010, 140062770* |
| Malignancy | *ICD-10 code: C00-C97* |
| Metastatic malignancy | *ICD-10 code: C77-C80* |
| COPD | *ICD-10 code: J41-J44* |
| Sepsis | *ICD-10 code: A021, A207, A227, A267, A327, A40, A400, A401, A402, A403, A408, A409, A41, A410, A411, A412, A413, A414, A415, A418, A419, A427, B377, O85, O883, P36, P360, P361, P362, P363, P364, P365, P368, P369* |
| Inflammation disease | *ICD-10 code: A02, A04, A05, A06, A08, A09, A15-17, A20, A32, A39, A40, A48, A54, A69, A74, A80-81, A83-87, B00-02, B05, B08, B15-20, B22, B25-26, B30, B33, B37-38, B58, B65, B91, B94-96, C86, E06, E79, F07, G00-05, G08-09, G21, G36-37, G51, G58, G61, G72, H00-01, H04-05, H10, H13, H15-16, H19-20, H22, H30, H32, H40, H44-46, H48, H60-62, H65-68, H70, H73, H75, H81, H83, H94, I01, I09, I30-33, I38-41, I67-68, I73, I77, I79, I80, I82-83, I88-89, J00-06, J09-18, J20-21, J30-32, J35, J37, J40-42, J67-69, J85, K04-05, K10-12, K14, K20-21, K23, K29, K35-37, K50-52, K62, K65, K67, K70-71, K73, K75, K80-81, K83, K85-86, L03-04, L13, L20-27, L30, L40, L44, L56, L58-59, L66, L71, L73, L81, L90, L95, L98, M00-01, M03, M05-09, M12-14, M30-31, M33, M35-36, M45-46, M49, M54, M60-61, M63, M65, M67-68, M70-73, M75-77, M79, M85-86, M88-90, M93-94, N00-07, N10-12,N30, N33-34, N37, N41, N45, N48-49, N51, N61, N70-74, N76-77, O22, O74, O87, O90-91, O98, P02, P23, P35, P38-39, P77-78, R09, R65, T39, T49, T82-85, U10* |
| Physical trigger | *ICD-10 code: A00-99, B00-99, D00-99, E00-99, G00-09, G40-47, G50-59, H60-69, I 26, I60-79, J00-22, J44, J46, J702, K25-29, K35, K55-56, K65, K67, K701, K71-72, K81-86, K91-92, L00-08, M00-36, M49-79, M86-90, N00-02, N10, N13-17, N20-23, N30, N34, N41, N44-45, N61, N70-77, O00-99, R00-99, S00-99, T00-99* |
| Emotional or unknown trigger | *A00-99, B00-99, E00-99, G00-09, G50-59, H60-69, I60-79, J00-22, K25-29, K35, K65, K67, K71-72, K81-86, L00-08, M00-36, M49-79, M86-90, N00-02, N10, N30, N34, N41, N44-45, N61, N70-77, O00-99, R00-99* |
| Heart failure | *ICD-10 code: I50* |
| Japan coma scale[3] | Japan coma scale is utilized to assess the level of consciousness in patients on admission. Patients are categorized into four groups for the analysis:  Alert [0]: Patients are fully responsive without any need for external stimuli.  Dizziness [1–3]: Patients are awake but experience slight disorientation or dizziness.  Somnolence [10–30]: Patients can be aroused by speech or respond to physical prompts by opening their eyes.  Coma [100–300]: Patients are unarousable by any form of stimuli. |
| Cardiopulmonary resuscitation | *ICD-10 code: I460*  **and/or**  *140010210, 150140010* |
| Intra-aortic balloon pump | *150148010, 150148110, 727610000, 727620000, 727630000* |
| Extracorporeal membrane  oxygenation or microaxial flow pump | Extracorporeal membrane oxygenation  *150262910, 150275710, 737160000, 737130000, 737130001, 737130000, 737140000, 737140001, 737140000, 737180000, 737210000, 737220000, 737240000, 737280000, 737250000, 710010779, 737260000, 737260000, 737230000, 737200000, 737270000, 737170000, 737190000, 738940000, 738950000, 738960000*  microaxial flow pump  In Japan, only the Impella® series are available.  *150395450, 150395550, 710011021* |
| Mechanical ventilation | *140009310, 140009450, 140009550, 140009650, 140009750, 140009850, 140009950, 140010050, 140010150, 140023510, 140023650, 140023750, 140023850, 140023950, 140024050, 140024150, 140024250, 140024350, 140030830, 140030930, 140031030, 140031130, 140031230, 140031330, 140031430, 140031530, 140031630, 140031730, 140039550, 140039650, 140039850, 140039950, 140063310, 140063410, 140063810, 140063950, 140064050, 140064150, 140064250, 140064350, 140064450, 140064550, 140064650, 140064750* |
| Oxygen therapy | *140005610* |
| Intensive care unit | *190024610, 190074610, 190116310, 190116410, 190139810, 190139910, 190140010, 190140110, 190140270, 190140370, 190174410, 190174510, 190174610, 190174710, 190174810, 190174910, 190207870, 190219270, 190223050, 190223150, 190223250, 190223350, 190223450, 190223550, 190223650, 190223750, 190223810, 190223910, 190224010, 190224110, 190227150, 190227250, 190227350, 190227450, 190227550, 190227650, 190227750, 190227850, 190230310, 190230410, 190230510, 190230610, 190230710, 190230810, 190230910, 190231010, 190233310, 190233410, 190233510, 190233610, 190235010, 190235110, 190235210, 190235310, 190236710, 190236810, 190236910, 190237010, 190246510, 190246610, 190246710, 190246810, 190246910, 190247010, 190247110, 190247210, 190247310, 190247470, 190247570, 190247670, 190717210, 190717310, 190717410, 190717510, 190717610, 190717710, 190727710, 190727810, 190727910, 190728010, 190728110, 190728210, 190728310, 190728410, 190737210, 190737310, 190737410, 190737510, 190737610, 190737710, 190737810, 190737910, 190746710, 190746810, 190746910, 190747010, 190747110, 190747210, 190747310, 190747410, 193001410, 193001510, 193001610, 193001710, 193005910, 193006010, 193006110, 193006210, 193006310, 193006410, 193010010, 193010110, 193010210, 193010310, 193010410, 193010510, 193010610, 193010710, 193012370, 193012470, 193014110, 193014210, 193014310, 193014410, 193014510, 193014610, 193014710, 193014810, 193014970, 193015070, 193015170, 193015270, 193301410, 193301510, 193301610, 193301710, 193307210, 193307310, 193307410, 193307510, 193307610, 193307710, 193310210, 193310310, 193310410, 193310510, 193310610, 193310710, 193310810, 193310910, 193315470, 193315570, 193317210, 193317310, 193317410, 193317510, 193317610, 193317710, 193317810, 193317910, 193318070, 193318170, 193318270, 193318370, 193501410, 193501510, 193501610, 193501710, 193507510, 193507610, 193507710, 193507810, 193507910, 193508010, 193508170, 193508270, 193511710, 193511810, 193511910, 193512010, 193512110, 193512210, 193512310, 193512410, 193521770, 193521870, 193532110, 193532210, 193532310, 193532410, 193532510, 193532610, 193532710, 193532810, 193532910, 193533010, 193533110, 193533210, 193533310, 193533410, 193533510, 193533610, 193533710, 193533810, 193533910, 193534010, 193534110, 193534210, 193534310, 193534410, 193534510, 193534610, 193534710, 193534810, 193534910, 193535010, 193535110, 193535210, 193535310, 193535410, 193535510, 193535610, 193535710, 193535810, 193535910, 193536010, 193536110, 193536210, 193536310, 193536410, 193536510, 193536610, 193536710, 193536810, 193536910, 193537010, 193537110, 193537210, 193537310, 193537410, 193537510, 193537610, 193537710, 193537810, 193537910, 193538010, 193538110, 193538210, 193538310, 193538410, 193538510, 193538610, 193538710, 193538810, 193538910, 193539010, 193539110, 193539210, 193558310, 193558410, 193558510, 193558610, 193558710, 193558810, 193558910, 193559010, 193559110, 193559210, 193559310, 193559410, 193559510, 193559610, 193559710, 193559810, 193559910, 193560010, 193560110, 193560210, 193560310, 193560410, 193560510, 193560610, 193560710, 193560810, 193560910, 193561010, 193561110, 193561210, 193561310, 193561410, 193561510, 193561610, 193561710, 193561810, 193561910, 193562010, 193562110, 193562210, 193562310, 193562410, 193562510, 193562610, 193562710, 193562810, 193562910, 193563010, 193563110, 193563210, 193563310, 193563410, 193563510, 193563610, 193563710, 193563810, 193563910, 193564010, 193564110, 193564210, 193564310, 193564410, 193564510, 193564610, 193564710, 193564810, 193564910, 193565010, 193565110, 193565210, 193565310, 193565410, 193575710, 193575810, 193575910, 193576010, 193576110, 193576210, 193576310, 193576410, 193576510, 193576610, 193576710, 193576810, 193576910, 193577010, 193577110, 193577210, 193577310, 193577410, 193582010, 193582110, 193582210, 193582310, 193582410, 193582510, 193582610, 193582710, 193582810, 193582910, 193583010, 193583110, 193583210, 193583310, 193583410, 193583510, 193583610, 193583710, 193588110, 193588210, 193588310, 193588410, 193588510, 193588610, 193588710, 193588810, 193588970, 193589070, 193589170, 193589270, 190296250, 190296350, 190297050, 190296450, 190296550, 190297150, 190295750, 190295850, 190295650, 190296650, 190296750, 190297250, 190296850, 190296950, 190297350, 190296050, 190296150, 190295950* |
| JCS certified hospital | Japan Cardiology Society (JCS) certified hospitals need more than one JCS board-certified cardiologist and 15 cardiovascular beds. |
| Inotrope | *ATC code: C01C* |
| Beta-blockers | *ATC code: C07A* |
| Anti-platelet drug | *ATC code: B01AC* |
| Proton pump inhibitor | *ATC code: A02BC* |
| Histamin-2 receptor antagonist | *ATC code: A02BA* |
| Dabigatran | *ATC code: B01AE07* |
| Rivaroxaban | *ATC code: B01AF01* |
| Apixaban | *ATC code: B01AF02* |
| Edoxaban | *ATC code: B01AF03* |
| Unfractionated heparin | *ATC code: B01AB01* |
| Low-molecular weight heparin | *ATC code: B01AB04-10, B01AB12* |

AIDS, Acquired Immune Deficiency Syndrome; ATC, Anatomical Therapeutic Chemical; ICD-10, the International Classification of Diseases, 10th revision.

Reference:

1. Mahoney FI, Barthel DW. FUNCTIONAL EVALUATION: THE BARTHEL INDEX. Md State Med J. 1965;14:61-5. PubMed PMID: 14258950.

2. Quan H, Li B, Couris CM, Fushimi K, Graham P, Hider P, et al. Updating and validating the Charlson comorbidity index and score for risk adjustment in hospital discharge abstracts using data from 6 countries. Am J Epidemiol. 2011;173(6):676-82. Epub 20110217. doi: 10.1093/aje/kwq433. PubMed PMID: 21330339.

3. Shigematsu K, Nakano H, Watanabe Y. The eye response test alone is sufficient to predict stroke outcome--reintroduction of Japan Coma Scale: a cohort study. BMJ Open. 2013;3(4). Epub 20130429. doi: 10.1136/bmjopen-2013-002736. PubMed PMID: 23633419; PubMed Central PMCID: PMCPMC3641437.
