# Supplementary material for: Comparison of treatment outcomes of direct oral anticoagulants and heparin for patients with Takotsubo cardiomyopathy: A nationwide cohort analysis
Source: PLoS One. 2025 Nov 13;20(11):e0336960. doi: 10.1371/journal.pone.0336960 (PMC12614514; doi:10.1371/journal.pone.0336960)
Supplement: S4 File — (DOCX) [file pone.0336960.s004.docx]

**S4. Difinitions of Outcomes**

|  |  |
| --- | --- |
| In-hospital mortality | In-hospital mortality was identified by meeting at least one of the following three criteria: (1) The patient's discharge outcome was recorded as “death”; (2) The patient died within 24 hours of admission; or (3) 'Death' was listed as the patient's post-discharge destination. |
| Ischemic event | Ischemic events were defined using specific diagnostic codes listed under “complications after admission”, which include cerebral infarction, transient ischemic attack, and arterial thrombosis. Each of these conditions was identified based on the following criteria. |
| Cerebral infarction | *ICD-10 code I63*  **and**  procedural codes for computed tomography or magnetic resonance imaging collected from the second day of admission onward.  *170011710, 170011810, 170011910, 170012070, 170012110, 170015550, 170022290, 170022390, 170022490, 170023010, 170023110, 170023210, 170027770, 170028610, 170028770, 170032870, 170033410, 170033970, 170034070, 170034910, 170036950, 170037670, 170037770, 170037870, 170038710, 170038810, 170038910, 170039010, 170039110, 170039210, 170040210, 170040310, 170040410, 170040510, 170040610, 170040710, 170040810, 170040910, 170041010, 170041110, 170041210, 170041310, 170015210, 170020110, 170020470, 170022110, 170023310, 170023410, 170023510, 170027870, 170033510, 170035010, 170035170, 170036270, 170037970, 170039410, 170039510, 170039610, 170039710, 170041410, 170041510, 170041610, 170041710, 170019950, 170700110, 170700610, 170701710, 170700510, 170701170, 170702010* |
| Transient ischemic attack | *ICD-10 code G45.9*  **and**  procedural codes for computed tomography or magnetic resonance imaging, as listed above, collected from the second day of admission onward. |
| Arterial thrombosis | *ICD-10 code H34*  **and**  procedural codes for computed tomography, magnetic resonance imaging (as listed above) or echocardiography (as listed below), collected from the second day of admission onward.  *160072510, 160072610, 160072750, 160072870, 160072910, 160147110, 160147210, 160150050, 160160410, 160161710, 160165010, 160189750, 160198810, 160205650, 160207270, 160207310, 160213010, 160072110, 160704510, 160704910, 160709110, 160704810, 160072450, 160072210* |
| Bleeding event | Bleeding events, including intracranial hemorrhage and gastrointestinal bleeding, were defined using specific diagnostic codes listed under “complications after admission” and associated procedure codes. |
| Intracranial hemorrhage | *ICD-10 code: I60, I61, I62*  **and**  procedural codes for computed tomography or magnetic resonance imaging, as listed above, collected from the second day of admission onwards. |
| Gastrointestinal bleeding | The definition of gastrointestinal bleeding was modified from criteria previously published by Fukasawa et al.[1]. These procedural codes were collected from the second day of admission onwards.  1. Endoscopic hemostasis  *150164850, 150263950, 140050910, 140010610, 150162910, 736610000, 736620000, 736630000*  2. Gastrointestinal endoscopy and thrombin on the same day  *160093410, 160093810, 160219210, 160226850, 160094110, 160094610, 160219310, 160094710, 160094810, 160094910*  **and**  *610406113, 610407240, 610407241, 613320194, 613320195, 620002482, 620002483, 620003540, 620003541, 620004483, 620004484, 620004485, 620008015, 620001650, 620001651, 620006459, 620006460, 620006461, 620006462, 620803312, 620803408, 620803603, 620803604, 620803703, 620803704, 660406069, 660406070, 660406130, 660406131, 660421093, 660421094, 660421095, 660421096, 660450004, 660450005, 663320017, 663320018, 663320019, 663320020, 663320030, 663320031, 663320032, 663320033, 663320034, 663320035, 663320036, 663320037, 663320038, 663320039, 663320042, 663320043, 663320044, 663320045, 663320046, 663320047, 663320048, 663320049, 663320050, 620006463, 660412032, 660470005, 660470006, 663320051, 620805001, 620805101, 660406006, 663320001, 663320004, 621518301, 621519801, 621519901, 621520001, 140050910, 620517902, 642450005, 642450164, 620518102, 662450001, 662450004*  3. The diagnostic code of esophagogastric varix or liver cirrhosis **and**  endoscopic injection sclerotherapy (EIS), endoscopic variceal ligation (EVL), or surgery for esophagogastric variceal bleeding  *ICD-10 code (standard disease code): I85.0, I86.4 (8845850, 8845851), K70.3 (8849244), K74.3 (8849298), K74.6 (8849260)*  **and**  *150136510, 150270150, 150136110, 150136210, 150136350, 150366910*  4. Transcatheter arterial embolization for GI bleeding  *ICD-10 code (standard disease code): I85.0, I86.4 (8845850, 8845851), K70.3(8849244), K74.3(8849298), K74.6(8849260), K22.8 (5308005), K25.0(5319011), K25.2, K25.4, K25.6, K26.0(8845123), K26.2, K26.4, K26.6, K27.0, K27.2, K27.4, K27.6, K28.0, K28.2, K28.4, K28.6, K29.0, K57.1(88455800), K57.3(8845742, 8845749, 8845763, 8845806, 8845814), K62.5, K92.0, K92.1, K92.2, K98.3*  **and**  *150360610, 150376810, 150360710* |
| Blood transfusion | *150224810, 150224910, 150286210, 150286310*  **and/or**  *620004647, 620004648, 620004663, 620004664, 620004671, 620004672, 620004673, 620004674, 620004675, 620004676, 620004677, 620004678, 620004679, 620004680, 620004681, 620004682, 620004687, 620004688, 620004692, 620004693, 620004744, 620004745, 621602201, 621602301, 621602401, 621602501, 621602601, 621602701, 621602801, 621602901, 621603001, 621609201, 621609301, 621609401, 621609501, 621609601, 621609701, 621609801, 621609901, 621610001, 621610701, 621772001, 621772101, 621772601, 621772701, 621772801, 621772901, 622190901, 622191001, 622191101, 622191201, 622191301, 622191401, 622191501, 622191601, 622191701, 622191801, 622191901, 622192001, 622192101, 622487001, 622487101, 640408044, 640408045, 640408046, 640421050, 640421051, 640421052, 640421053, 640421054, 640421055, 640421056, 640421057, 640421058, 640421059, 640421060, 640421061, 640421062, 640421073, 640421074, 640421075, 640421076, 640421077, 640421078, 640421079, 640421080, 646340037, 646340048, 646340223, 646340226, 646340242, 646340243, 646340244, 646340245, 646340285, 646340286, 646340292, 646340293, 646340294, 646340295, 646340297, 646340298, 646340299, 646340300, 646340301, 646340307, 646340308, 646340309, 646340310, 646340313, 646340314, 646340315, 646340316, 646340507, 646340508, 646340509, 646340511, 646340512, 646340514, 646340515, 646340516, 646340517, 646340482, 646340483* |
| The length of hospital stay | The number of days from admission to discharge. |
| Total hospitalization cost | The cost included all medical care, the bundled payment for each hospitalization was calculated according to the codes in the International Classification of Diseases 10th revision (ICD-10) and the coefficient for each facility. The costs are presented with an exchange rate of 120 yen to 1 USD. |

Reference:

1. Fukasawa T, Seki T, Nakashima M, Kawakami K. Comparative effectiveness and safety of edoxaban, rivaroxaban, and apixaban in patients with venous thromboembolism: A cohort study. J Thromb Haemost. 2022;20(9):2083-97. Epub 20220707. doi: 10.1111/jth.15799. PubMed PMID: 35748327.
