# Supplementary material for: Comparison of treatment outcomes of direct oral anticoagulants and heparin for patients with Takotsubo cardiomyopathy: A nationwide cohort analysis
Source: PLoS One. 2025 Nov 13;20(11):e0336960. doi: 10.1371/journal.pone.0336960 (PMC12614514; doi:10.1371/journal.pone.0336960)
Supplement: S1 Table — Data are presented as n (%). Age and BMI are presented as mean (standard deviation). Continuous variables were compared using the t-test and categorical variables were compared using the chi-squared test. BMI, body mass index; COPD, chronic obstructive pulmonary disease; IABP, intra-aortic balloon pump; ECMO, extracorporeal membrane oxygenation; JCS, Japan Circulation of Society. (DOCX) [file pone.0336960.s005.docx]

**S1 Table. Patient characteristics before and after propensity score matching**

|  | Before matching | | | After matching | | |
| --- | --- | --- | --- | --- | --- | --- |
|  | DOAC | Heparin | SMD | DOAC | Heparin | SMD |
| n | 530 | 4283 |  | 442 | 442 |  |
| Age, years, mean (SD) | 78.1 (9.4) | 74.4 (11.2) | 0.353 | 77.4 (9.7) | 77.5 (9.8) | 0.01 |
| Female sex | 421 (79.4) | 3574 (83.4) | 0.103 | 358 (81.0) | 356 (80.5) | 0.011 |
| BMI, kg/m2, mean (SD) | 21.3 (3.8) | 20.9 (3.7) | 0.082 | 21.1 (3.8) | 21.1 (3.5) | 0.018 |
| Smoker | 86 (16.2) | 781 (18.2) | 0.053 | 76 (17.2) | 74 (16.7) | 0.012 |
| Barthel Index |  |  | 0.167 |  |  | 0.051 |
| totally dependent [0-20] | 187 (41.3) | 1749 (49.4) |  | 158 (42.5) | 165 (45.0) |  |
| partially dependent [25-95] | 104 (23.0) | 666 (18.8) |  | 80 (21.5) | 77 (21.0) |  |
| independent [100] | 162 (35.8) | 1122 (31.7) |  | 134 (36.0) | 125 (34.1) |  |
| Charlson score |  |  | 0.097 |  |  | 0.089 |
| 0 | 122 (23.0) | 1018 (23.8) |  | 101 (22.9) | 99 (22.4) |  |
| 1 | 222 (41.9) | 1638 (38.2) |  | 181 (41.0) | 197 (44.6) |  |
| 2 | 132 (24.9) | 1080 (25.2) |  | 114 (25.8) | 99 (22.4) |  |
| 3 or more | 54 (10.2) | 547 (12.8) |  | 46 (10.4) | 47 (10.6) |  |
| Hypertension | 364 (68.7) | 2478 (57.9) | 0.226 | 288 (65.2) | 288 (65.2) | 0 |
| Diabetes mellitus | 109 (20.6) | 830 (19.4) | 0.03 | 89 (20.1) | 85 (19.2) | 0.023 |
| Dyslipidemia | 202 (38.1) | 1507 (35.2) | 0.061 | 164 (37.1) | 145 (32.8) | 0.09 |
| Atrial fibrillation | 282 (53.2) | 301 (7.0) | 1.165 | 194 (43.9) | 194 (43.9) | 0 |
| Pulmonary embolism | 4 (0.8) | 15 (0.4) | 0.055 | 4 (0.9) | 5 (1.1) | 0.023 |
| Deep vein thrombosis | 14 (2.6) | 36 (0.8) | 0.138 | 14 (3.2) | 14 (3.2) | 0 |
| Cerebrovascular disease | 10 (1.9) | 73 (1.7) | 0.014 | 10 (2.3) | 7 (1.6) | 0.049 |
| Severe kidney disease | 0 (0.0) | 52 (1.2) | 0.157 | 0 (0.0) | 0 (0.0) | 0 |
| Malignancy | 28 (5.3) | 232 (5.4) | 0.006 | 26 (5.9) | 21 (4.8) | 0.05 |
| COPD | 8 (1.5) | 123 (2.9) | 0.093 | 7 (1.6) | 9 (2.0) | 0.034 |
| Sepsis | 1 (0.2) | 32 (0.7) | 0.082 | 1 (0.2) | 1 (0.2) | 0 |
| Inflammation disease | 127 (24.0) | 1206 (28.2) | 0.096 | 112 (25.3) | 114 (25.8) | 0.01 |
| Trigger |  |  | 0.171 |  |  | 0.019 |
| Physical | 174 (32.8) | 1758 (41.0) |  | 149 (33.7) | 153 (34.6) |  |
| Emotional or unknown | 356 (67.2) | 2525 (59.0) |  | 293 (66.3) | 289 (65.4) |  |
| Heart failure | 243 (45.8) | 1753 (40.9) | 0.099 | 203 (45.9) | 195 (44.1) | 0.036 |
| Japan coma scale |  |  | 0.103 |  |  | 0.074 |
| alert [0] | 442 (84.7) | 3480 (82.0) |  | 372 (85.5) | 368 (84.8) |  |
| dizziness [1-3] | 61 (11.7) | 553 (13.0) |  | 47 (10.8) | 51 (11.8) |  |
| somnolence [10-30] | 12 (2.3) | 98 (2.3) |  | 9 (2.1) | 11 (2.5) |  |
| coma [100-300] | 7 (1.3) | 111 (2.6) |  | 7 (1.6) | 4 (0.9) |  |
| Cardiopulmonary resuscitation | 5 (0.9) | 58 (1.4) | 0.039 | 3 (0.7) | 3 (0.7) | 0 |
| IABP | 1 (0.2) | 127 (3.0) | <0.001 | 1 (0.2) | 1 (0.2) | 0 |
| ECMO or microaxial flow pump | 0 (0.0) | 21 (0.5) | 0.205 | 0 (0.0) | 0 (0.0) | 0 |
| Mechanical ventilation | 23 (4.3) | 503 (11.7) | 0.275 | 23 (5.2) | 29 (6.6) | 0.058 |
| Oxygen therapy | 270 (50.9) | 2453 (57.3) | 0.127 | 231 (52.3) | 233 (52.7) | 0.009 |
| Intensive care unit | 78 (14.7) | 860 (20.1) | 0.142 | 67 (15.2) | 65 (14.7) | 0.013 |
| JCS certified hospital | 215 (40.6) | 1887 (44.1) | 0.071 | 174 (39.4) | 165 (37.3) | 0.042 |
| Inotrope | 87 (16.4) | 1074 (25.1) | 0.215 | 75 (17.0) | 75 (17.0) | 0.000 |
| Beta-blocker | 209 (39.4) | 941 (22.0) | 0.386 | 147 (33.3) | 145 (32.8) | 0.01 |
| Anti-platelet drug | 245 (46.2) | 2098 (49.0) | 0.055 | 213 (48.2) | 210 (47.5) | 0.014 |
| Warfarin | 1 (0.2) | 309 (7.2) | 0.379 | 1 (0.2) | 2 (0.5) | 0.039 |
| Proton pump inhibitor | 285 (53.8) | 1923 (44.9) | 0.178 | 223 (50.5) | 225 (50.9) | 0.009 |
| Histamin-2 receptor antagonist | 26 (4.9) | 359 (8.4) | 0.14 | 23 (5.2) | 21 (4.8) | 0.021 |
| Dabigatran | 25 (4.7) |  |  | 20 (4.5) |  |  |
| Rivaroxaban | 108 (20.4) |  |  | 91 (20.6) |  |  |
| Apixaban | 190 (35.8) |  |  | 147 (33.3) |  |  |
| Edoxaban | 209 (39.4) |  |  | 186 (42.1) |  |  |
| Unfractionated heparin |  | 4283 (100.0) |  |  | 442 (100.0) |  |
| Low-molecular weight heparin |  | 2 (0.0) |  |  | 0 (0.0) |  |

Data are presented as n (%). Age and BMI are presented as mean (standard deviation). Continuous variables were compared using the t-test and categorical variables were compared using the chi-squared test. BMI, Body mass index; COPD, chronic obstructive pulmonary disease; IABP, intra-aortic balloon pump; ECMO, extracorporeal membrane oxygenation; JCS, Japan Circulation of Society
