# Supplementary material for: Comparison of treatment outcomes of direct oral anticoagulants and heparin for patients with Takotsubo cardiomyopathy: A nationwide cohort analysis
Source: PLoS One. 2025 Nov 13;20(11):e0336960. doi: 10.1371/journal.pone.0336960 (PMC12614514; doi:10.1371/journal.pone.0336960)
Supplement: S2 Table — DOAC, direct oral anticoagulant; NA; not available; RR, incidence rate ratio. (DOCX) [file pone.0336960.s006.docx]

**S2 Table. Clinical outcomes in the matched cohort**

|  | Before matching | | After matching | | | |
| --- | --- | --- | --- | --- | --- | --- |
|  | DOAC | Heparin | DOAC | Heparin | IRR |  |
| n | 530 | 4283 | 442 | 442 | [95% CI] | p |
| Primary outcome |  |  |  |  |  |  |
| In-hospital mortality | 21 (4.0) | 174 (4.1) | 19 (4.3) | 16 (3.6) | 1.44 [0.50-4.12] | 0.5 |
|  |  |  |  |  |  |  |
| Secondary outcomes |  |  |  |  |  |  |
| Ischemic events | 6 (1.1) | 59 (1.4) | 4 (0.9) | 13 (2.9) | 0.37 [0.12-1.11] | 0.076 |
| Cerebral infarction | 6 (1.1) | 45 (1.1) | 4 (0.9) | 11 (2.5) | 0.44 [0.14-1.36] | 0.16 |
| Transient ischemic attack | 0 (0.0) | 2 (0.0) | 0 (0.0) | 0 (0.0) | NA | NA |
| Arterial thrombosis | 0 (0.0) | 12 (0.3) | 0 (0.0) | 2 (0.5) | NA | NA |
| Bleeding events | 1 (0.2) | 24 (0.6) | 1 (0.2) | 2 (0.5) | 0.60 [0.05-7.28] | 0.69 |
| Intracranial hemorrhage | 0 (0.0) | 11 (0.3) | 0 (0.0) | 1 (0.2) | NA | NA |
| Gastrointestinal bleeding | 1 (0.2) | 13 (0.3) | 1 (0.2) | 1 (0.2) | 1.21 [0.10-14.76] | 0.88 |
|  |  |  |  |  |  |  |
| Blood transfusion | 10 (1.9) | 239 (5.6) | 9 (2.0) | 27 (6.1) | 0.40 [0.21-0.79] | 0.008 |

DOAC, direct oral anticoagulant; IRR, incidence rate ratio, NA; not available
