# Supplementary material for: Comparison of treatment outcomes of direct oral anticoagulants and heparin for patients with Takotsubo cardiomyopathy: A nationwide cohort analysis
Source: PLoS One. 2025 Nov 13;20(11):e0336960. doi: 10.1371/journal.pone.0336960 (PMC12614514; doi:10.1371/journal.pone.0336960)
Supplement: S3 Table — DOAC, direct oral anticoagulant. (DOCX) [file pone.0336960.s007.docx]

**S3 Table. Length of hospital stay and hospitalization costs in the matched cohort**

|  | Before matching | | After matching | | |
| --- | --- | --- | --- | --- | --- |
|  | DOAC | Heparin | DOAC | Heparin | p |
| n | 530 | 4283 | 442 | 442 |  |
| Length of hospital stay | 11 (8-17) | 13 (9-19) | 11 (8-17) | 13 (9-19) | 0.002 |
| Total hospitalization cost | 5190 (4000-7623) | 6130 (4569-9013) | 5338 (4000-7661) | 6030 (4549-8106) | 0.017 |

DOAC, direct oral anticoagulant
