# Supplementary material for: Comparison of treatment outcomes of direct oral anticoagulants and heparin for patients with Takotsubo cardiomyopathy: A nationwide cohort analysis
Source: PLoS One. 2025 Nov 13;20(11):e0336960. doi: 10.1371/journal.pone.0336960 (PMC12614514; doi:10.1371/journal.pone.0336960)
Supplement: S4 Table — Categorical variables are presented as n (%) before IPTW and as weighted percentages after IPTW. Age and BMI are presented as means (standard deviation). BMI, body mass index; COPD, chronic obstructive pulmonary disease; DOAC, direct oral anticoagulant; ECMO, extracorporeal membrane oxygenation; IABP, intra-aortic balloon pump; IPTW, inverse probability of treatment weighting; JCS, Japan Circulation Society; SMD, standardized mean difference. (DOCX) [file pone.0336960.s008.docx]

**S4 Table. Patient characteristics in the atrial fibrillation cohort before and after IPTW**

|  | Before IPTW | | | After IPTW | | |
| --- | --- | --- | --- | --- | --- | --- |
|  | DOAC | Heparin | SMD | DOAC | Heparin | SMD |
| n | 282 | 301 |  |  |  |  |
| Age, years, mean (SD) | 78.3 (9.2) | 77.5 (9.2) | 0.086 | 78.3 (9.2) | 77.6 (9.3) | 0.08 |
| Female sex | 218 (77.3) | 249 (82.7) | 0.136 | 77.3 | 78.1 | 0.019 |
| BMI, kg/m2, mean (SD) | 21.3 (3.7) | 21.0 (3.6) | 0.068 | 21.3 (3.7) | 21.1 (3.4) | 0.061 |
| Smoker | 47 (16.7) | 47 (15.6) | 0.029 | 16.7 | 15.2 | 0.039 |
| Barthel Index |  |  | 0.171 |  |  | 0.038 |
| totally dependent [0-20] | 102 (41.1) | 121 (49.6) |  | 39.4 | 37.6 |  |
| partially dependent [25-95] | 58 (23.4) | 49 (20.1) |  | 24.1 | 24.3 |  |
| independent [100] | 88 (35.5) | 74 (30.3) |  | 36.5 | 38.1 |  |
| Charlson score |  |  | 0.078 |  |  | 0.039 |
| 0 | 65 (23.0) | 61 (20.3) |  | 23.0 | 22.5 |  |
| 1 | 128 (45.4) | 141 (46.8) |  | 45.4 | 45.8 |  |
| 2 | 70 (24.8) | 75 (24.9) |  | 24.8 | 24.0 |  |
| 3 or more | 19 (6.7) | 24 (8.0) |  | 6.7 | 7.6 |  |
| Hypertension | 200 (70.9) | 169 (56.1) | 0.311 | 70.9 | 69.7 | 0.026 |
| Diabetes mellitus | 44 (15.6) | 42 (14.0) | 0.046 | 15.6 | 14.4 | 0.034 |
| Dyslipidemia | 99 (35.1) | 77 (25.6) | 0.208 | 35.1 | 33.0 | 0.045 |
| Pulmonary embolism | 0 (0.0) | 0 (0.0) | NA | 0.0 | 0.0 | NA |
| Deep vein thrombosis | 2 (0.7) | 3 (1.0) | 0.031 | 0.7 | 0.7 | 0.003 |
| Cerebrovascular disease | 4 (1.4) | 3 (1.0) | 0.039 | 1.4 | 0.5 | 0.098 |
| Severe kidney disease | 0 (0.0) | 2 (0.7) | 0.116 | 0.0 | 0.0 | 0 |
| Malignancy | 13 (4.6) | 9 (3.0) | 0.085 | 4.6 | 4.4 | 0.01 |
| COPD | 5 (1.8) | 4 (1.3) | 0.036 | 1.8 | 1.0 | 0.068 |
| Sepsis | 0 (0.0) | 2 (0.7) | 0.116 | 0.0 | 0.0 | 0 |
| Inflammation disease | 53 (18.8) | 78 (25.9) | 0.172 | 18.8 | 20.0 | 0.03 |
| Trigger |  |  | 0.133 |  |  | 0.025 |
| Physical | 81 (28.7) | 105 (34.9) |  | 28.7 | 29.9 |  |
| Emotional or unknown | 1 (0.4) | 2 (0.7) |  | 71.3 | 70.1 |  |
| Heart failure | 139 (49.3) | 157 (52.2) | 0.057 | 49.3 | 51.0 | 0.034 |
| Japan coma scale |  |  | 0.188 |  |  | 0.022 |
| alert [0] | 236 (85.8) | 237 (80.1) |  | 85.8 | 86.5 |  |
| dizziness [1-3] | 33 (12.0) | 44 (14.9) |  | 12.0 | 11.6 |  |
| somnolence [10-30] | 4 (1.5) | 8 (2.7) |  | 1.5 | 1.3 |  |
| coma [100-300] | 2 (0.7) | 7 (2.4) |  | 0.7 | 0.6 |  |
| Cardiopulmonary resuscitation | 4 (1.4) | 2 (0.7) | 0.074 | 1.4 | 1.1 | 0.03 |
| IABP | 1 (0.4) | 8 (2.7) | 0.19 | 0.4 | 0.4 | 0.012 |
| ECMO or microaxial flow pump | 0 (0.0) | 0 (0.0) | NA | 0.0 | 0.0 | NA |
| Mechanical ventilation | 11 (3.9) | 49 (16.3) | 0.42 | 3.9 | 4.7 | 0.039 |
| Oxygen therapy | 151 (53.5) | 176 (58.5) | 0.099 | 53.5 | 55.3 | 0.035 |
| Intensive care unit | 43 (15.2) | 51 (16.9) | 0.046 | 15.2 | 13.2 | 0.059 |
| JCS certified hospital | 129 (45.7) | 138 (45.8) | 0.002 | 45.7 | 41.5 | 0.086 |
| Inotrope | 48 (17.0) | 92 (30.6) | 0.322 | 17.0 | 18.6 | 0.042 |
| Beta-blocker | 126 (44.7) | 87 (28.9) | 0.332 | 44.7 | 42.4 | 0.046 |
| Anti-platelet drug | 130 (46.1) | 133 (44.2) | 0.038 | 46.1 | 44.8 | 0.026 |
| Warfarin | 0 (0.0) | 44 (14.6) | 0.585 | 0.0 | 0.0 | 0 |
| Proton pump inhibitor | 155 (55.0) | 129 (42.9) | 0.244 | 55.0 | 51.7 | 0.065 |
| Histamin-2 receptor antagonist | 14 (5.0) | 32 (10.6) | 0.213 | 5.0 | 5.8 | 0.038 |
| Dabigatran | 14 (5.0) |  |  | 5.0 |  |  |
| Rivaroxaban | 68 (24.1) |  |  | 24.1 |  |  |
| Apixaban | 114 (40.4) |  |  | 40.4 |  |  |
| Edoxaban | 87 (30.9) |  |  | 30.9 |  |  |
| Unfractionated heparin |  | 301 (100.0) |  |  | 100.0 |  |
| Low-molecular weight heparin |  | 0 (0.0) |  |  | 0.0 |  |

Categorical variables are presented as n (%) before IPTW and as weighted percentages after IPTW. Age and BMI are presented as means (standard deviation). BMI, body mass index; COPD, chronic obstructive pulmonary disease; DOAC, direct oral anticoagulant; ECMO, extracorporeal membrane oxygenation; IABP, intra-aortic balloon pump; IPTW, inverse probability of treatment weighting; JCS, Japan Circulation Society; SMD, standardized mean difference.
