# Supplementary material for: Comparison of treatment outcomes of direct oral anticoagulants and heparin for patients with Takotsubo cardiomyopathy: A nationwide cohort analysis
Source: PLoS One. 2025 Nov 13;20(11):e0336960. doi: 10.1371/journal.pone.0336960 (PMC12614514; doi:10.1371/journal.pone.0336960)
Supplement: S5 Table — All outcomes are presented as n (%) before IPTW and as weighted percentages after IPTW. The RRs and CIs were not calculated if one group had no events for a specific outcome. CI, confidence interval; DOAC, direct oral anticoagulant; IPTW, inverse probability of treatment weighting; NA, not applicable; RR, risk ratio. (DOCX) [file pone.0336960.s009.docx]

**S5 Table. Clinical outcomes in the atrial fibrillation cohort**

|  | Before IPTW | | After IPTW | |  |  |
| --- | --- | --- | --- | --- | --- | --- |
|  | DOAC | Heparin | DOAC | Heparin | RR [95% CI] | p |
| n | 282 | 301 |  |  |  |  |
| Primary outcome |  |  |  |  |  |  |
| In-hospital mortality | 7 (2.5) | 13 (4.3) | 2.5 | 4.3 | 0.60 [0.21-1.70] | 0.34 |
|  |  |  |  |  |  |  |
| Secondary outcomes |  |  |  |  |  |  |
| Ischemic events | 5 (1.8) | 7 (2.3) | 1.8 | 4.8 | 0.38 [0.12-1.22] | 0.11 |
| Cerebral infarction | 5 (1.8) | 7 (2.3) | 1.8 | 4.8 | 0.38 [0.12-1.22] | 0.11 |
| Transient ischemic attack | 0 (0.0) | 0 (0.0) | 0 | 0 | NA | NA |
| Arterial thrombosis | 0 (0.0) | 0 (0.0) | 0 | 0 | NA | NA |
| Bleeding events | 1 (0.4) | 1 (0.3) | 0.4 | 0.1 | 4.79 [0.30-76.95] | 0.27 |
| Intracranial hemorrhage | 0 (0.0) | 0 (0.0) | 0 | 0 | NA | NA |
| Gastrointestinal bleeding | 1 (0.4) | 1 (0.3) | 0.4 | 0.1 | 4.79 [0.30-76.95] | 0.27 |
| Blood transfusion | 3 (1.1) | 21 (7.0) | 1.1 | 5.7 | 0.19 [0.05-0.71] | 0.014 |

All outcomes are presented as n (%) before IPTW and as weighted percentages after IPTW. The RRs and CIs were not calculated if one group had no events for a specific outcome. DOAC, direct oral anticoagulant; IPTW, inverse probability of treatment weighting; NA, not applicable; RR, risk ratio; CI, confidence interval
